# Rapid simultaneous determination of indacaterol maleate and glycopyrronium bromide in inhaler capsules using a validated stability-indicating monolithic LC method

**DOI:** 10.1186/s13065-017-0264-6

**Published:** 2017-05-04

**Authors:** Sahar Zayed, Fathalla Belal

**Affiliations:** 1 Unit of Drug Analysis, Faculty of Pharmacy, University of Mansoura, Mansoura, 35516 Egypt; 2 Pharmaceutical Analytical Chemistry Department, Faculty of Pharmacy, University of Mansoura, Mansoura, 35516 Egypt

**Keywords:** Indacaterol maleate, Glycopyrronium bromide, HPLC, Monolithic column, Stability indicating, Inhaler capsules

## Abstract

**Background:**

Chronic obstructive pulmonary disease (COPD) is a major cause of morbidity and mortality worldwide. A combination of indacaterol maleate with glycopyrronium bromide has recently been approved as a once-daily maintenance therapy in patients with COPD. The very low dose (μg level/capsule) renders the analysis of such products challenges. This study reports for the first time about HPLC method for the quality control of such combination and it is a stability indicating at the same time.

**Results:**

A rapid, simple, precise and reproducible HPLC method was developed and validated for simultaneous determination of indacaterol maleate and glycopyrronium bromide using tenoxicam as an internal standard. The chromatographic separation was achieved on an onyx monolithic C18 column (100 × 4.6 mm) using a mobile phase consisting of acetonitrile and 30 mM phosphate buffer (pH 3.5) (30:70, v/v), run at a flow rate of 2 mL/min with UV detection at 210 nm. The total analysis time was less than 3 min. The HPLC method was validated for linearity, limits of detection and quantitation, precision, accuracy, system suitability and robustness. Calibration curves were obtained in the concentration ranges of 1–44 µg/mL for indacaterol maleate and 0.5–20 µg/mL for glycopyrronium bromide. Stability tests were done through exposure of the analyte solution for different stress conditions and the results indicate no interference of degradants with HPLC method.

**Conclusions:**

The method was successfully applied for the quantitative analysis of indacaterol maleate and glycopyrronium bromide both individually and in a combined pharmaceutical inhaler capsules to support the quality control and to assure the therapeutic efficacy of the two drugs. The simple procedure involved in sample preparation and the short run-time added the important property of high throughput to the method.

**Graphical abstract:**
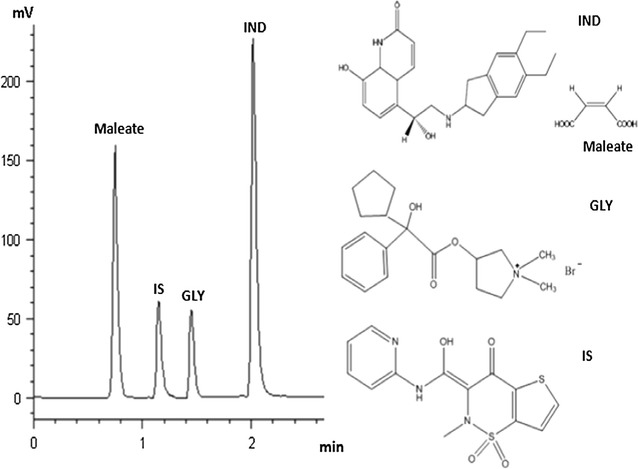
Chemical structures and representative HPLC chromatogram of indacaterol maleate (IND; 22 μg/mL), glycopyrronium bromide (GLY; 10 μg/mL) and tenoxicam (IS, 15μg/mL) in commercial capsules.

## Background

**Fig. 1:**
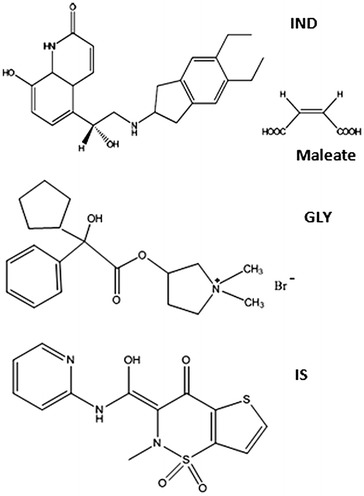
Chemical structures of indacaterol maleate (IND), glycopyrronium bromide (GLY) and tenoxicam (IS)

Chronic obstructive pulmonary disease (COPD) is a prevalent lung disease caused by chronic airway and pulmonary inflammation which lead to progressive airflow limitation. Long-acting inhaled bronchodilators are the recommended first-line maintenance treatment for COPD [1]. Indacaterol maleate (IND), 5-{(1R)-2-[(5,6-diethyl-2,3-dihydro-1H-inden-2-yl)amino]-1-hydroxyethyl}-8-hydroxy-2(1H)-quinolinone maleate, is the first ultra-long-acting β2-agonist bronchodilator that has been approved by the U.S. Food and Drug Administration (FDA) in July 2011 [2]. Glycopyrronium bromide (GLY), 3-[(Cyclopentylhydroxyphenylacetyl) oxy]-1,1-dimethyl-pyrrolidinium bromide, a new long-acting muscarinic antagonist was approved in Europe in 2012 for maintenance bronchodilator treatment in patients with moderate to severe COPD [3]. Recently, the combination of IND and GLY as a dual-bronchodilator therapy is the preferred choice for COPD treatment because of its powerful bronchodilator effects and a simple once-daily inhalation regimen [4]. The chemical structures of both drugs are shown in Fig. 1.

Few analytical methods have been reported in the literature for the individual determination of IND or GLY. These methods include: spectrophotometry [5, 6], HPLC [7], GC [8], spectrofluorometry [5] and HPLC–MS methods [9–14]. IND is not cited in any pharmacopoeia while GLY is cited in European Pharmacopoeia (E.P.), British Pharmacopoeia (B.P) and United States Pharmacopoeia (U.S.P.). However, no HPLC method for simultaneous determination of IND and GLY in combined dosage forms has been reported so far.

High-performance liquid chromatography (HPLC) is usually the analytical method of choice for pharmaceutical quality control [15]. It is a demand of the time to develop high-throughput HPLC methods with high efficiency. Monolithic HPLC columns are considered as one of the modern approaches for fast analysis and an interesting alternative to particulate-based HPLC columns [16]. Due to their rigid and porous structure, they enable higher rates of mass transfer at lower pressure drops as well as high efficiencies even at elevated flow rates [17]. This enhances the speed of the separation process and reduces backpressure and unspecific binding without sacrificing resolution [18, 19].

The present study describes, for the first time, a rapid, simple and stability-indicating HPLC method using a monolithic column with UV detection. The proposed HPLC method allowed the quantitative determination of the two drugs in their commercial inhaler capsules with satisfactory accuracy and precision. Thus, the developed method can be used for routine analysis laboratories and quality control purposes.

## Experimental

### Apparatus

Chromatographic analyses were carried out using a Shimadzu Prominence HPLC system (Shimadzu Corporation, Japan) with a LC-20 AD pump, DGU-20 A5 degasser, CBM-20A interface, a column oven (CTO-20A) and SPD-20A UV–VIS detector with 20 μL injection loop. An ultrasonicator from Merck L-7612 and a pH meter from Hanna (USA) were used. UV lamp short wavelength 254 nm (Vilber Lournate 220 V 50 Hz, Marne-la-Vallee Cedex, France) was used in the UV-degradation study.

### Materials and reagents

All the chemicals used were of analytical reagent grade, and the solvents were of HPLC grade. Indacaterol maleate and glycopyrronium bromide reference substances were kindly provided by Novartis (Basel, Switzerland). Tenoxicam (TNX) as internal standard and maleic acid were obtained from Sigma Chemicals. Inhaler capsules containing 110 µg of IND and 50 µg of GLY/capsule (Ultibro^**®**^ Breezhaler^**®**^), 150 µg of IND/capsule (Onbrez Breezhaler^**®**^) and 50 µg of GLY/capsule (Seebri^**®**^ Breezhaler^**®**^) were obtained from commercial sources. Acetonitrile and methanol were purchased from Sigma-Aldrich (Germany). Orthophosphoric acid (85% w/v) was obtained from Riedel-deHaën (Sleeze, Germany). Hydrochloric acid (32% w/v), hydrogen peroxide (10% w/v), sodium hydroxide and sodium dihydrogen phosphate were obtained from Adwic Co. (Cairo, Egypt). High purity distilled water was used in the study.

### Chromatographic conditions

An Onyx Monolithic C18, 100 × 4.6 mm (Phenomenex, Torrance, California, USA) thermostatted at 35 °C was used in this study. The mobile phase consisting of acetonitrile-30 mM phosphate buffer adjusted to pH 3.5 with orthophosphoric acid (30:70, v/v) was filtered through a 0.45 μm Millipore membrane filter under vacuum. The flow rate was 2.0 mL/min and UV detection was set at 210 nm.

### Standard solutions

Stock solutions of 200 µg/mL of IND, GLY, and 500 µg/mL TNX (IS) were individually prepared in methanol. These stock solutions were further diluted with the same solvent and then with the mobile phase as appropriate to obtain the working standard solutions. The stock solutions were stored at 4 °C, protected from light.

### Construction of calibration graphs

Aliquots of the suitable drug stock or working standard solutions were transferred into a series of 10-mL volumetric flasks so that the final concentrations were in the range of 1–44 μg/mL for IND and 0.5–20 μg/mL for GLY. A constant 300 μL TNX stock solution was added (final concentration of 15 μg/mL) and the volumes were diluted to 10 mL with the mobile phase. The peak area ratio (peak area of the studied drug/peak area of TNX) was plotted versus the final concentration of each drug in μg/mL to get the calibration graph. Alternatively, the corresponding regression equations were derived.

### Preparation of sample solutions

Ten capsules from each formulation were emptied and the contents were weighted. A quantity of the powder equivalent to 440 μg of IND and 200 μg of GLY (Ultibro^**®**^ Breezhaler^**®**^), 450 µg of IND (Onbrez Breezhaler^**®**^) and 200 µg of GLY (Seebri^**®**^ Breezhaler^**®**^) was transferred into individual 10.0 mL volumetric flasks, sonicated with the mobile phase for 10 min and then the solution was completed to volume with the mobile phase. For analysis, an appropriate aliquot from the prepared sample solutions, spiked with 300 µL TNX stock solution, was diluted to 10 mL using the same solvent. All solutions were filtered through a 0.45 µm membrane filter before injection to the HPLC system. The nominal contents of the capsules were calculated using either the calibration graphs or the corresponding regression equations.

### Preparation of the degradation products

1 mL aliquots of each of the stock solutions of IND and GLY were transferred into a series of screw capped glass vials followed by 2 mL of distilled water, 0.1 M HCl, 0.1 M NaOH or 10% hydrogen peroxide (H_2_O_2_). The solutions were heated in a thermostatically controlled water bath at 80 °C for 1 h. At the specified time, the contents of the vials were cooled, and solutions under acidic and alkaline treatment were neutralized with NaOH and HCl solutions, respectively. Photo degradation was induced by exposing the samples to near ultraviolet (254 nm) light for 8 h. The solutions were transferred into a series of 10 mL volumetric flasks. Then, 300 µL of the IS solution was added, and the volumes were completed with the mobile phase. Solutions were mixed well and triplicate 20 µL injections were made for each sample. The samples were analyzed against a freshly prepared control standard solution.

## Results and discussion

The simultaneous separation and quantification of IND and GLY within the minimum analysis time and the maximum resolution and efficiency is the main objective of this study. The polarity of GLY and IND differ greatly, as GLY is less lipophilic than IND and their log P were found −1.2 and 3.31, respectively. The large difference in lipophilicity between GLY and IND posed a challenge in the development of the separation. The monolithic column was selected which yielded the advantage to optimize the separation of such drugs by utilizing an isocratic run. During our preliminary experiments, we tried several combinations of the mobile phase composition and pH in order to obtain the optimum separation.

## Method development and optimization

### Choice of detection wavelength

Proper choice of the detection wavelength is crucial for the sensitivity of the method. The detection of IND and GLY was attempted at different wavelengths including 210, 220, 230 and 254 nm; 210 nm was selected as the optimum detection wavelength allowing the detection of the two drugs and their degradation products with high sensitivity.

### Effect of pH and ionic strength of the buffer

The effect of changing the pH of phosphate buffer solution was tested from 3.0 to 7.0. The increase in the pH from 4.0 to 7.0, caused loss of the peak sharpness and peak symmetry with a slight increase in retention time of both drugs. The peak shapes for the two drugs were sufficiently symmetrical only for pH value below 4.0. Little change is observed between pH 3.0 and 4.0. So, pH 3.5 was found to be optimal. Studying the ionic strength of phosphate buffer (10–50 mM) revealed no significant effect on the separation process or the retention time of the two drugs. Hence, 30 mM phosphate buffer was used as the aqueous phase in this study.

### Variation of type and concentration of the organic modifier

Two different organic solvents, methanol and acetonitrile were used. It was found that acetonitrile resulted in better sensitivity, shorter analysis time, and improvement in the peak shape compared with methanol. The influence of the amount of acetonitrile in the mobile phase was examined from 20 to 40%. When acetonitrile content was increased to 40%, the retention time of the two drugs was decreased with overlapping of their peaks. Whereas the use of 20% acetonitrile caused a delay in the elution with a decrease in the number of theoretical plates. A concentration of 30% acetonitrile was found to be the best compromise between selectivity and analysis time.

### Effect of column temperature and flow rate

The influence of column temperature was examined in the range from 25 to 45 °C. As expected at higher temperature, the retention of IND and GLY decreased, but the resolution between them decreased simultaneously. A temperature of 35 °C was found to give the best compromise to improve the repeatability between runs and reduce the analysis time.

The effect of the flow rate on the separation of the two drugs was also investigated. Good separation of IND and GLY with good peaks’ shape and minimum retention times (<3 min) was obtained at a flow rate 2.0 mL/min.

### Internal standard selection

For choosing a suitable internal standard, several drugs were tested. Tenoxicam was chosen as the best IS, giving a symmetrical peak; well separated from the two drugs and all the degradation products.

**Fig. 2:**
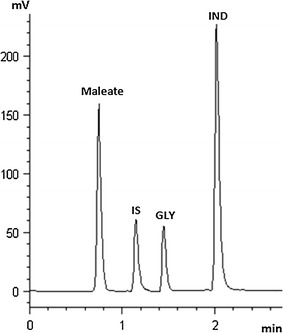
Representative HPLC chromatogram of indacaterol maleate (IND; 22 µg/mL), glycopyrronium bromide (GLY; 10 µg/mL) and tenoxicam (IS; 15 µg/mL) in commercial capsules. Chromatographic conditions: monolithic C18 column (100 mm, 4.6 mm id); mobile phase: acetonitrile-30 mM sodium phosphate of pH 3.5 (30:70, v/v); flow rate 2.0 mL/min; column temperature of 35 °C; detection: 210 nm

Under the mentioned chromatographic conditions highly symmetrical and sharp peaks of GLY and IND were obtained at retention times of 1.47 and 2.18 min, respectively. The method was able to separate IND from its acidic counter ion (maleate). The peak due to counter ion was confirmed by injecting maleic acid solution and also by spiking in the drug solution to prove the elution at 0.71 min. A typical chromatogram of a commercial sample of IND and GLY capsule is shown in Fig. 2.

### Method validation

The method was validated according to the International Conference on Harmonization (ICH) guidelines [20, 21]. Different validation characteristics were investigated as follows:

### Linearity

A linear relationship was established by plotting the peak area ratio against the drug concentration. The concentration ranges were found to be linear in the range of 1–44 and 0.5–20 μg/mL of IND and GLY, respectively. The values of the determination coefficient (R^2^) calculated were 0.9999 (y = 1.345x + 0.061) for IND and 0.9998 (y = 0.183x + 0.059) for GLY, indicating the linearity of the analytical curves for the method, where x is concentration and y is the peak area ratio.

### Limit of detection (LOD) and Limit of quantification (LOQ)

The LOD and LOQ for IND and GLY were determined based on signal-to-noise ratio of 3 and 10, respectively. The baseline noise was measured in a blank experiment in the region of retention time of IND and GLY using chromatographic software. It was found that for IND, the LOD and LOQ values were 0.06 and 0.16 µg/mL (RSD = 0.82%), respectively and for GLY, the LOD and LOQ values were 0.12 and 0.34 µg/mL (RSD = 0.74%), respectively.

### Precision

The precision of the method was evaluated as repeatability and intermediate precision. Repeatability was examined by threefold analyses of two preparations of 22 µg/mL of IND and 10 µg/mL of GLY in 1 day. The RSD on the peak areas of these six determinations was not more than 0.46%. Intermediate precision was also determined for three consecutive days. The RSD on the peak areas was not more than 1.13% suggesting that the proposed method is suitable for simultaneous analysis of IND and GLY in combined dosage forms.

### Accuracy

Accuracy of the proposed method was determined by the standard addition method on the dosage form to which known amounts of IND and GLY standards have been added at different concentrations (IND: 5.5, 15, 22 µg/mL; GLY: 2.5, 5, 10 µg/mL). The determination was carried out using three replicates at each concentration level. The accuracy was determined as percent recovery of amount of analyte added to the sample. As shown in Table 1, the method was accurate within the desired range.

**Table 1:** Accuracy of the proposed HPLC method

Formulation	Concentration taken	Concentration added (µg/mL)		Accuracy %^a^	
Ultibro^®^ Breezhaler^®^	11, 5 µg/mL	IND	GLY	IND	GLY
		5.5	2.5	99.81	100.19
		15	5	100.34	101.02
		22	10	100.72	100.35
Mean % ± SD				100.29 ± 0.46	100.52 ± 0.44
%RSD				0.46	0.44
Onbrez Breezhaler^®^	15 µg/mL	IND		IND	
		5.5		101.09	
		15		100.21	
		22		99.78	
Mean % ± SD				100.36 ± 0.67	
%RSD				0.67	
Seebri^®^ Breezhaler^®^	5 µg/mL		GLY		GLY
			2.5		99.42
			5		100.37
			10		100.14
Mean % ± SD					99.98 ± 0.49
%RSD					0.49

^a^Each result is the average of three separate determinations

### Robustness

The robustness of the developed method was investigated by evaluating the influence of small deliberate variations in experimental parameters like flow rate (±0.1 mL/min), detection wavelength (±2 nm), buffer pH (±0.2) and acetonitrile content (±2%). Resolution between GLY/IND, theoretical plates and assay % of the two drugs were determined for each modified condition. Table 2 shows the experiments performed for robustness evaluation. Therefore, it can be seen that these minor changes did not greatly affect the method performance.

**Table 2:** Robustness data for the proposed method

Variation	Resolution	Theoretical plates		Assay (%)	
		IND	GLY	IND	GLY
28% ACN	4.81	4785	4658	99.86	100.23
32% ACN	3.57	4924	4826	100.71	100.96
pH 3.3	4.13	4875	4732	100.49	100.91
pH 3.7	4.32	4817	4715	100.37	100.35
Flow (1.9 mL)	4.42	4858	4715	100.35	100.34
Flow (2.1 mL)	3.91	4894	4737	100.52	100.87
Wavelength 208 (nm)	–	4889	4734	100.73	101.14
Wavelength 212 (nm)	–	4862	4719	100.29	100.12
Temperature 33 °C	4.33	4861	4712	100.3	100.24
Temperature 37 °C	4.14	4884	4741	100.61	100.81
Without variation	4.25	4870	4725	100.43	100.62

### System suitability

System suitability tests were performed to ensure that the HPLC system and the developed method are capable of providing quality data, based on USP 32 requirements [22]. The system suitability test carried out presented the following results: RSD values of 0.19 and 0.24% for the retention times, 0.78 and 1.12% for the peak areas, and 0.14 and 0.28% for the tailing factors, for GLY and IND, respectively. The number of theoretical plates was about 4725 and 4870 for GLY and IND, respectively. The experimental results showed that the parameters tested were within the acceptable range (RSD < 2.0%), indicating that the method is suitable for the analysis intended.

### Stability of solutions

The stability of standard working solutions as well as sample solutions in the diluting solvent (mobile phase) was examined and no chromatographic changes were observed within 8 h at room temperature and 48 h at 4 °C. Also, the stock solutions prepared in HPLC-grade methanol were stable for at least 2 weeks when stored refrigerated at 4 °C. Retention times and peak areas of the drugs remained unchanged and no significant degradation was observed during these periods.

### Forced-degradation and stability-indicating aspects

**Fig. 3:**
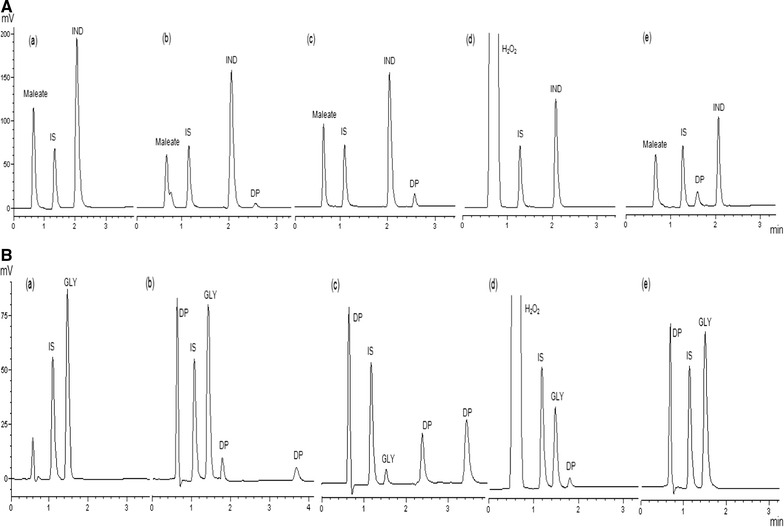
HPLC chromatograms of **A** indacaterol maleate (IND; 20 μg/mL) and **B** glycopyrronium bromide (GLY; 20 μg/mL) after (*a*) neutral hydrolysis (*b*) acidic hydrolysis; (*c*) alkaline hydrolysis; (*d*) oxidation and (*e*) exposition to UV light. *IS* internal standard (15 µg/mL), *DP* degraded products

The presence of degradants and impurities in pharmaceutical formulations can result in changes in their chemical, pharmacological, and toxicological properties affecting their efficacy and safety of the drugs [23]. Therefore, the adoption of stability-indicating methods is always required to control the quality of pharmaceuticals during and after the production. This greatly contributes to the possibility of improving drug safety [24]. After acid, alkaline, and neutral hydrolysis of IND, the content of the drug decreased (31.1, 33.5, and 3.8%, respectively). An additional peak was also observed in alkaline and acidic conditions. Under oxidative conditions, IND remained 61.4% intact without any additional peak. Under the photolytic conditions, IND content exhibited a 48.7% decrease of the area after 8 h, and one degradation product was detected. Figure 3A shows the chromatograms of the IND forced degradation studies. Under the acidic hydrolysis, GLY content exhibited a decrease of its area (17.6%) after 1 h in 0.1 M HCl solution, and three additional peaks were detected. Under the basic hydrolysis, nearly 95.8% of the GLY was degraded after 1 h in 0.1 M NaOH solution, and three additional peaks were detected. GLY was stable under neutral hydrolysis for 1 h. Just one additional peak was also detected in the oxidative condition with a 49.5% decrease of the GLY peak. For the photolytic condition, 21.7% of the GLY was degraded after 8 h and one degradation product was detected. The chromatograms of the forced degradation studies of GLY are represented in Fig. 3B.

### Application of the proposed method to pharmaceutical analysis

The proposed method was successfully applied for the determination of IND and GLY both individually and in combined dosage capsules formulations. No interfering peaks were observed in the recorded chromatograms indicating that there is no interference effect resulting from excipients used in the production of capsules. The results obtained are accurate and precise as indicated by the excellent percentage recovery (Table 3). The assay results obtained has shown that the method is suitable for the quality control analysis of IND and GLY.

**Table 3:** Determination of IND and GLY in commercial inhaler capsules

Formulation	Labelled amount		Mean found		Recovery^a^ (%)	
	IND	GLY	IND	GLY	IND	GLY
Ultibro^®^ Breezhaler^®^	110	50	110.73	50.58	100.67 ± 0.35	101.16 ± 0.47
Onbrez Breezhaler^®^	150	–	150.54	–	100.36 ± 0.26	–
Seebri^®^ Breezhaler^®^	–	50	–	50.25	–	100.49 ± 0.56

^a^Each result is the average of three separate determinations

## Conclusion

A simple, rapid, and accurate LC method was developed for the simultaneous determination of IND and GLY in pharmaceutical inhaler capsules using monolithic column. The LC method was validated and demonstrated good linearity, precision, accuracy and specificity without any interference from the excipients and degradation products. The proposed method could be adopted in quality control laboratories for the analysis of these two drugs individually or in combination.

## Abbreviations

IND: indacaterol maleate
GLY: glycopyrronium bromide
COPD: chronic obstructive pulmonary disease
HPLC: high performace liquid chromatography
ICH: International Conference on Harmonization
LOQ: limit of quantification
LOD: limit of detection
